# α-lipoic acid modulates prostate cancer cell growth and bone cell differentiation

**DOI:** 10.1038/s41598-024-54479-x

**Published:** 2024-02-22

**Authors:** K. M. Abdullah, Gunjan Sharma, Simran Takkar, Jyoti B. Kaushal, Ramesh Pothuraju, Bandana Chakravarti, Surinder K. Batra, Jawed A. Siddiqui

**Affiliations:** 1 Department of Biochemistry and Molecular Biology, University of Nebraska Medical Center, Omaha, NE 68198 USA; 2 Cancer Research Program, Rajiv Gandhi Centre for Biotechnology, Thiruvananthapuram, Kerala 695014 India; 3 Department of Endocrinology, Sanjay Gandhi Postgraduate Institute of Medical Sciences, Lucknow, Uttar Pradesh 226014 India; 4 Fred and Pamela Buffett Cancer Center, University of Nebraska Medical Center, Omaha, NE 68198 USA; 5 Eppley Institute for Research in Cancer and Allied Diseases, University of Nebraska Medical Center, Omaha, NE 68198 USA; 6 Department of Biochemistry and Molecular Biology, Fred and Pamela Buffett Cancer Center, University of Nebraska Medical Center, Omaha, NE 68198-5870 USA

**Keywords:** Prostate cancer, Bone modulation, Osteoblasts, Osteoclasts, α-LA, Reactive oxygen species (ROS), HIF-1α, p-JNK, Bone metastases, Cancer prevention

## Abstract

Prostate cancer (PCa) progression leads to bone modulation in approximately 70% of affected men. A nutraceutical, namely, α-lipoic acid (α-LA), is known for its potent anti-cancer properties towards various cancers and has been implicated in treating and promoting bone health. Our study aimed to explore the molecular mechanism behind the role of α-LA as therapeutics in preventing PCa and its associated bone modulation. Notably, α-LA treatment significantly reduced the cell viability, migration, and invasion of PCa cell lines in a dose-dependent manner. In addition, α-LA supplementation dramatically increased reactive oxygen species (ROS) levels and HIF-1α expression, which started the downstream molecular cascade and activated JNK/caspase-3 signaling pathway. Flow cytometry data revealed the arrest of the cell cycle in the S-phase, which has led to apoptosis of PCa cells. Furthermore, the results of ALP (Alkaline phosphatase) and TRAP (tartrate-resistant acid phosphatase) staining signifies that α-LA supplementation diminished the PCa-mediated differentiation of osteoblasts and osteoclasts, respectively, in the MC3T3-E1 and bone marrow macrophages (BMMs) cells. In summary, α-LA supplementation enhanced cellular apoptosis via increased ROS levels, HIF-1α expression, and JNK/caspase-3 signaling pathway in advanced human PCa cell lines. Also, the treatment of α-LA improved bone health by reducing PCa-mediated bone cell modulation.

## Introduction

Prostate cancer (PCa) is one of the most common cancers globally, making it a severe threat to men’s health and the second most significant cause of death from cancer worldwide^1,2^. The slow and subtle progression of PCa is frequently accompanied by delayed tumor detection, which is a possible cause of metastasis. One of the most common sites of PCa metastasis is bone due to genetic and epigenetic alterations during tumor initiation^3,4^. Bone metastasis is a severe clinical concern due to its association with poor prognosis and reduced survival rate^5,6^. Bone metastasis is associated with a substantial decline in the 5-year survival rate, plummeting from 60% in patients without skeletal metastasis to a dismal 3% with bone involvement^7^. The pain associated with bone metastasis and other pathological characteristics indicates the incurability of disease in most cases^3^. Over the past several decades, bone-targeted therapies have been used to interrupt the crosstalk between PCa and bone cells, holding potential as a therapeutic strategy for advanced PCa patients with bone metastasis^8,9^.

Among the diverse treatment options for bone diseases, micronutrients and nutraceuticals have gained attention for their cost-effectiveness, safety, and ability to target specific molecular events in affected cells and tissues. One such nutraceutical, α-lipoic acid (α-LA), has gained popularity due to its antioxidant properties and crucial role in mitochondrial bioenergetics^10,11^. It has been reported that many human dietary components like kidney, liver, and muscle mass contain appreciable amounts of α-LA. At the same time, vegetables and fruits have lower amounts distributed to various tissues after ingestion^12^. α-LA has shown cytotoxic and anti-proliferative effects in several cancers, including breast, ovarian, colorectal, and lung cancer^13–18^. Subsequently, an essential reason for α-LA to be a vital nutraceutical is its chemoprotective potential^19^. Additionally, it has been investigated that α-LA not only restrains the activity of bone-resorbing osteoclasts but also supports bone-forming osteoblasts^11^.

Furthermore, α-LA can exert both pro- and antioxidant effects, depending on the underlying physiologic and metabolic state^20^. In cancer cells, α-LA has been found to induce apoptosis and inhibit proliferation through various pathways, including the regulation of reactive oxygen species (ROS) levels^21^. Hypoxia-inducible factor (HIF), particularly HIF-1α, is a key regulator in response to cellular stressors, and excessive ROS levels can influence its expression. JNK, a downstream factor of HIF-1α, modulates tumor cell apoptosis or proliferation^22^. Recent studies highlighted the importance of ROS accumulation in activation of the JNK/caspase-3 pathway to induce apoptosis in PCa cells^23^. In PCa, α-LA affects the antioxidant system of the cells and may be related to the compensatory changes in their antioxidant defense system. It was concluded that α-LA induces apoptosis by a pro-oxidant mechanism triggered by an escalated uptake of mitochondrial substrates in oxidizable form^24^. Although the possible cytotoxic effect of α-LA was shown in PCa cells, the exact mechanism behind the pro-oxidant effect of α-LA is poorly understood. Therefore, despite the vast knowledge about the multiple effects of α-LA, the possibility that α-LA can prevent PCa mediated bone cells modulation has not been researched to the best of our knowledge. Therefore, the present study aims to elucidate the mechanism involved in the anti-neoplastic potential of α-LA in PCa bone metastases. By investigating the cytotoxicity in 22Rv1 and C4-2B cells under α-LA treatment, we have observed a significant increase in apoptosis due to intracellular ROS production. Additionally, we identified that the HIF-1α signaling pathway plays an essential role during this process. Moreover, the present study highlighted the beneficial effect of α-LA in PCa-mediated bone loss, as there was a significant decline in the PCa-mediated differentiation of bone cells. These findings contribute to the growing research on nutraceuticals as an alternative therapeutic approach in managing advanced PCa and bone metastasis.

## Material and methods

### Cell culture and cell lines

Human PCa cell lines (22Rv1, C4-2B) and preosteoblast cell line (MC3T3-E1) were obtained from ATCC (Rockville, MD, USA), maintained in RPMI and α-MEM media, respectively, supplemented with 1% penicillin–streptomycin antibiotics (100 μg/ml) and 10% fetal bovine serum (FBS) at 37 °C with 5% CO_2_ in the humidifier atmosphere. The fresh media were replaced every 2 days, and culture conditions were maintained as described earlier^25,26^. All cell lines were tested routinely to determine mycoplasma contamination and STR profile. For the preparation of condition media (CM), 2 × 10^6^ cells of 22Rv1 were grown in 100 mm tissue culture dish for 24 h in 10% RPMI medium. The day before CM collection, cells were washed with phosphate buffer saline (PBS) and incubated with 1% RPMI media for 48 h.

### Cell proliferation and colony-forming assay

The cell viability and proliferation were measured using 3-(4,5-dimethylthiazol-2-yl)-2,5-diphenyltetrazolium bromide (MTT) and colony formation assays, respectively. For MTT, 2500 cells/wells were seeded in a 96-well plate overnight in an RPMI medium supplemented with 10% FBS. The next day, cells were treated with α-LA at different concentrations (50, 100, 200, 300, 500, 1000, and 2000 μM) for 48 h after aspirating the culture media. After completion of 48 h, MTT was added and subsequently kept in the incubator for 2 h at 37 °C. After 2 h, MTT formazan crystals were dissolved in dimethyl sulfoxide, and the absorbance was measured at 570 nm. Cell viability was measured by calculating absorbance according to its relative changes.

For colony formation assay, 1000 cells/well were seeded in a 6-well plate in RPMI supplemented with 10% FBS. After 24 h, cells were treated with 500 μM of α-LA, and untreated cells were used as a control group. Fresh medium was subsequently added to both groups every 48 h for 14 days. Later, cells were washed with PBS and fixed by 0.5% crystal violet as previously described^27^. The number of colonies was counted and quantified using the Image-J processing tool.

### Wound healing assay

For wound healing assays, 1 × 10^6^ cells of both 22Rv1 and C4-2B were seeded in 35 mm culture dish, followed by the treatment with α-LA (500 µM). After 48 h, viable cells were counted and seeded in a 6-well plate in 10% RPMI medium and maintained in a growth medium until 89–90% confluency. Then, using a sterile pipette tip, a straight scratch was made on monolayer cells, and subsequently, detached cells were removed using PBS. Further, migrating cells were monitored using an inverted microscope (EVOS FL Auto) at 0, 24, and 48 h, as described earlier^28^.

### Transwell migration assay

For invasion assay, 1 × 10^6^ cells were seeded in a 35 mm culture dish and treated with 500 μM α-LA for 48 h. The untreated cells served as the control. At the end of the incubation period, viable cells were counted and seeded on the top of the Boyden chamber (8 μm Corning^®^ BioCoat™ Matrigel^®^ Invasion Chamber) in the growth medium deprived of serum for 48 h; 20% FBS in RPMI medium was added in the lower side of a 6-well plate. After 48 h, the cells invaded in the lower chamber were then stained and fixed with Diff-Quick stain; further invaded cells across different fields were counted in both the control and treated groups. The average number of migrated cells per field was calculated and plotted using the GraphPad software. Each experiment was done in triplicates.

### Western blot analysis

Total cell lysates were collected in RIPA lysis buffer containing 150 mM NaCl, 0.1% sodium dodecyl sulfate, 50 mM Tris–HCl, 0.5% sodium deoxycholate, along with inhibitors of phosphatase and protease. The collected lysates were syringe passed and centrifuged for 25 min at 15,000×*g*, 4 °C, and then quantified by Bio-Rad DC Protein Assay kit. The protein samples (20–40 µg per well) were resolved by SDS-PAGE, followed by immunoblotting on the PVDF membrane. Membranes were blocked for 1 h in 5% non-fat dry milk dissolved in PBS containing 0.1% tween-20 (PBST). Further, membranes were immunolabelled with primary antibodies overnight at 4 °C. Details of antibodies used in the experiments are provided in Table 1. The next day, membranes were washed three times with PBST probed with respective secondary antibodies (1:5000 dilutions) at room temperature for 1 h, and immunolabelling was performed using chemiluminescence ECL reagent as described earlier^29^.

**Table 1:** Antibodies used in this study.

Antibodies	Source	Identifier
GPx4, WB: 1:1000	Cell signaling technology	Cat#52455S
p-Rb (Ser-807/811), WB: 1:1000	Cell signaling technology	Cat#9308
p21, WB: 1:1000	Cell signaling technology	Cat#2947
β-actin, WB-1:500	Santa Cruz	Cat#sc-517582
Catalase, WB-1:2000	Abcam	Cat#ab16731
SOD1, WB-1:300	Santa Cruz	Cat#sc-101523
SOD2, WB-1:300	Santa Cruz	Cat#sc-137254
Bax, WB: 1:1000	Cell signaling technology	Cat#5023
BcL-xL, WB: 1:1000	Cell signaling technology	Cat#2764
HIF1-α, WB: 1000	Cell signaling technology	Cat#3716
E-cadherin, WB: 1:1000	Cell signaling technology	Cat#3195
p-JNK, WB: 1:1000	Cell signaling technology	Cat#9251
cyclin E, WB: 1:1000	Cell signaling technology	Cat#4129
N-cadherin, WB: 1:1000	Cell signaling technology	Cat# 13116
Vimentin, WB: 1:1000	Cell signaling technology	Cat#5741
Cleaved caspase 3: 1:1000	Invitrogen	Cat#700182

### RNA isolation and real-time PCR analysis

Total RNA from different experiments was isolated using a Qiagen RNA isolation kit. RNA (1 µg) was used for cDNA synthesis using TaqMan^®^ Reverse Transcription Reagents and random hexamers. For quantitative real-time PCR, SYBR^®^ Green Master Mix (Roche) was used, and cycle conditions included 10 min at 95 °C, 95 °C for 15 s for 40 cycles, and 1 min at 58 °C. The mRNA expression profile of different genes was calculated as described earlier^30^. The expression levels were normalized using β-actin, and the data is represented in fold changes compared to control samples^31^. The primers utilized in this study are mentioned in Table 2.

**Table 2:** Primer sequences of various genes.

Gene	Species	Forward primer	Reverse primer
ALP	Mouse	ATCTTTGGTCTGGCTCCCATG	TTTCCCGTTCACCGTCCAC
Osteocalcin	Mouse	GCAATAAGGTAGTGAACAGACTCC	GTTTGTAGGCGGTCTTCAAGC
RunX2	Mouse	AGTCCCAACTTCCTGTGCTCC	CGGTAACCACAGTCCCATCTG
Col1a1	Mouse	CCTTCATGTCCAAGCAGGA	GCGCCGGAGTCTGTTCACTA
TRAP	Mouse	CACTCCCACCCTGAGATTTGTG	ACGGTTCTGGCGATCTCTTTGC
NFATc1	Mouse	TCCACAGTCATTTGCTCTGC	TCCAGCAGGAGGCTATGTG
Carbonic Anhydrase	Mouse	TGGTTCACTGGAACACCAAA	AGCAAGGGTCGAAGTTAGCA
c-fos	Mouse	AATGGTGAAGACCGTGTCAGGA	CCCTTCGGATTCTCCGTTTCT
Cathepsin K	Mouse	CAGCAGAGGTGTGTACTATG	GCGTTGTTCTTATTCCGAGC
β-actin	Mouse	TCCTCCTGAGCGCAAGTACTCT	CGGACTCATCGTACTCCTGCTT

### Cell cycle analysis

Approximately 1 × 10^6^ cells/well of both (C4-2B and 22Rv1) cell lines were seeded in RPMI media deprived of serum and treated with 500 μM of α-LA for 48 h. The untreated cells were used as a control group. After completion of the incubation period, cells were fixed with 70% ethanol overnight at 4 °C. The following day, fixed cells were washed in PBS and then, for 1 h, stained with Telford reagent at 4 °C as described previously^30^. The stained cells were then analyzed through flow cytometry FACS to determine the phases of the cell cycle.

### Apoptosis assay

C4-2B and 22Rv1 (1 × 10^6^ cells/well) were seeded in a 6-well plate overnight in 10% RPMI growth medium for apoptosis assay. The next day, treatment of 500 μM α-LA for 48 h. After the completion of 48 h, untreated (control) and treated cells were trypsinized, washed twice with PBS, and suspended in the 1X-FACS binding buffer. Next, both the groups were incubated for 15 min with propidium iodide (PI) and Annexin-V/Cy^TM^5- in the dark. Then, stained cells were determined by flow cytometry analysis.

### ROS assessment assay

Approximately 2 × 10^3^ cells/well of C4-2B and 22Rv1 were counted and seeded in a 96-well plate for 24 h in a 10% RPMI growth medium. At the end of incubation time, seeded cells were incubated with 500 μM of α-LA. Subsequently, at the end of 48 h, cells were exposed for 30 min to DCFH-DA. Next, intracellular levels of ROS were analyzed by measuring fluorescence using a microplate reader (SYNERGY neo2, BioTek) at 485 nm (excitation) and 535 nm (emission). Subsequently, imaging was done using a microscope (EVOS FL Auto).

### Differentiation of osteoclast from bone marrow macrophages of the mouse (BMMs)

Bone marrow cells were isolated from 4-week-old C57BL/6J mice as described earlier^32^. In brief, bones were separated from the femur and tibia. The bone marrow cavity was flushed using a 25-gauge needle and α-MEM. Next, isolated BMMs were cultured in 10% FBS containing α-MEM along with mouse receptor activator of NF-kappaB ligand (mRANKL) and mouse macrophage colony-stimulating factor (mMCSF) as described previously^33^. This culture was exposed to 20% and 50% CM from 22Rv1 cells and 50% CM along with 100 nM (nM) of α-LA. The medium was changed after every 2 days. Subsequently, after 72 h, mRNA analysis was performed using qRT-PCR. At the end of 7 days, Tartrate-resistant acid phosphatase (TRAP) was performed to determine the differentiated osteoclasts^32^.

### TRAP staining

After the completion of osteoclast differentiation, osteoclasts were observed under a light microscope, and cells were fixed for 30 min using 4% paraformaldehyde dissolved in PBS at room temperature and further using a Leukocyte acid phosphatase (TRAP) kit (387-A; Sigma–Aldrich) differentiated osteoclasts were stained according to the instructions. Images of multinucleated TRAP-positive cells were captured with an EVOS FL auto microscope^33^.

### Pit resorption

BMMs were seeded onto pre-coated inorganic bone biomaterial surface (Corning Osteo Assay Surface Polystyrene) 1 × 8 Stripwell™ Microplate and stimulated with RANKL (100 ng/ml) and treated with 22Rv1 CM 20, 50% and 50% along with α-LA (100 nM) for 7–8 days. As for pit formation, media and drugs were removed, and 5% sodium hypochlorite was added for 5 min and washed with dH_2_O. Then, the bone resorption/pit formation was photographed from each well with an EVOS FL auto microscope^34^.

### Differentiation of osteoblasts

MC3T3-E1 cells were cultured in α-MEM media supplemented with 10% FBS and 1% penicillin–streptomycin. Cells (2 × 10^3^ cells/well) were seeded in 96-well plate after cells reached 80–90% confluency. After 24 h, seeded cells were subjected to treatment with osteoblast differentiation media (10 mmol·L^−1^ β-glycerophosphate, 10% α-MEM, and 50 mg·mL^−1^ ascorbic acid) alone, 20%, 40%, and 50% CM from 22Rv1 cells and 50% CM along with 100 nM of α-LA for 2 days in osteoblast differentiation media. The untreated cells were used as the control group. For alkaline phosphatase (ALP) activity, p-nitrophenyl phosphate was used as the substrate and quantified at 405 nm, and ALP staining was photographed, as described earlier^35^.

### Statistical analysis

Student’s t-test or one-way ANOVA was applied to evaluate differences using the GraphPad InStat software program (GraphPad Software, Inc.). P values < 0.05 were considered to indicate statistical significance.

## Results

### α-Lipoic acid treatment decreases cell viability and S-phase cell cycle arrest

**Figure 1:**
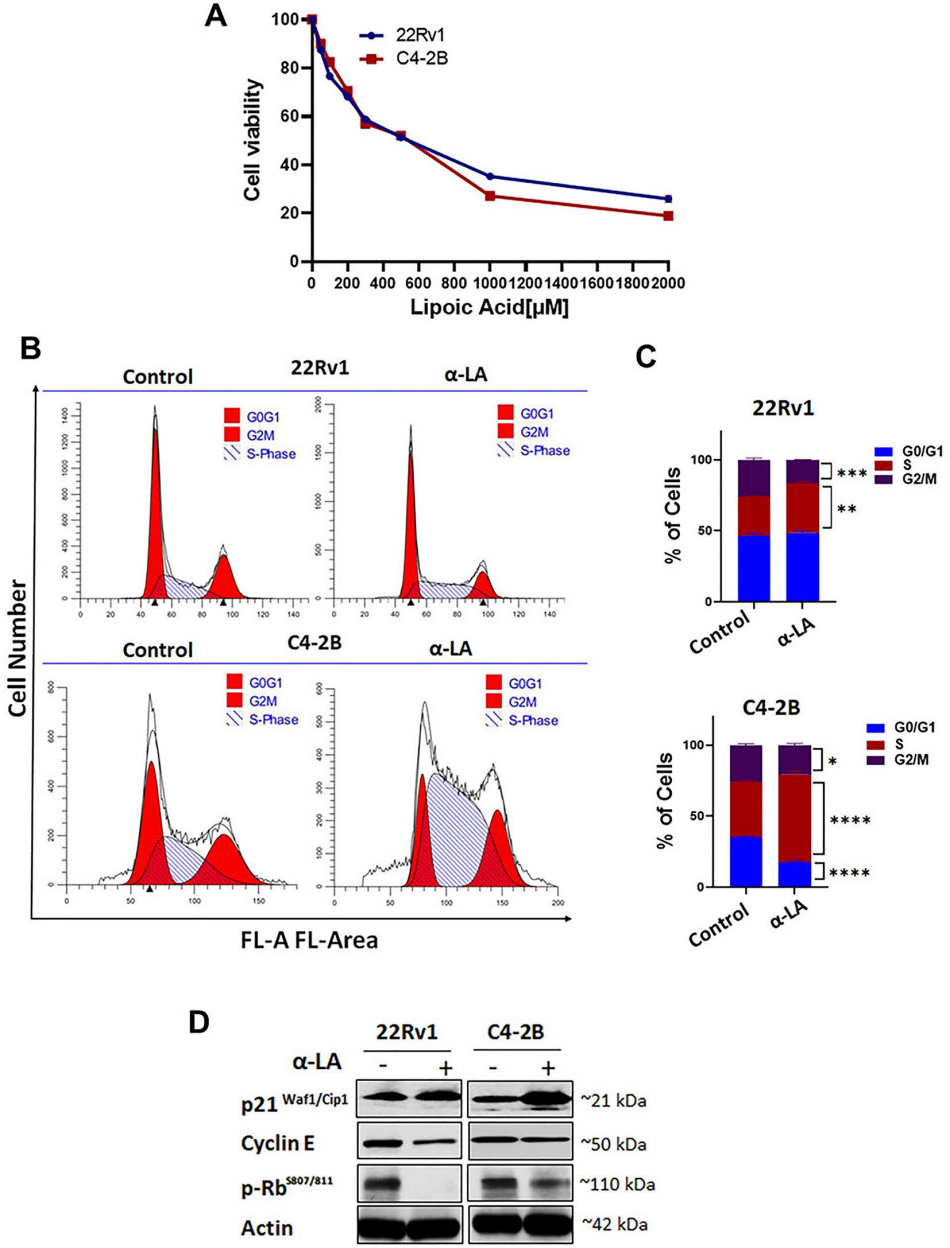
Effect of increasing concentrations of α-LA on PCa cell viability determined by MTT assay (**A**). Cells were treated with various concentrations of α-LA for 48 h, and loss of viability was measured by MTT assay. Values are expressed as mean ± SEM. The cell cycles of 22Rv1 and C4-2B cells were treated with 500 µM of α-LA. (**B**) DNA content in different cell cycle phases was assessed by propidium iodide staining of control and treated cells, followed by FACS analysis. Cell cycle analysis showed that after α-LA treatment, cells were arrested at the S phase. (**C**) Quantitative analysis of DNA content was shown as mean ± SEM (n = 3). (**D**) Western blot analysis of 22Rv1 and C4-2B performed after α-LA treatment reveals that the cell cycle arrest facilitates via upregulation of p21 protein and downregulation of cyclin E and pRb protein. Statistical significance was calculated using two-way ANOVA. ****p < 0.0001; ***p < 0.0002; **p < 0.001; *p < 0.05.

To assess the impact of α-LA on cell viability, 22Rv1 and C4-2B cell lines were treated with various concentrations of α-LA for 48 h, and cell viability was evaluated using the MTT assay. α-LA treatment resulted in significantly lower cell viability compared to the control in a dose-dependent manner with an IC_50_ of 500 µM for both the cell lines, as shown in Fig. 1A. We employed flow cytometry to examine the DNA content of various cell cycle phases, and interestingly, it was found that α-LA treatment (500 µM) showed a S-phase arrest in both 22Rv1 and C4-2B cell lines (Fig. 1B,C). Cell cycle arrest is caused by α-LA therapy in hepatoma cells and is associated with an increased expression of the cyclin-dependent kinase inhibitors p21^Cip^ and p27^Kip^^36^. Therefore, we analyzed the expression profiles of two critical regulators of cell cycle progression, p21 and cyclin E, after 48 h of treatment with α-LA. Concurrently, there was an upregulation of p21 expression, indicating the involvement of cyclin-dependent cell cycle arrest due to α-LA treatment (Fig. 1D). These findings suggest that α-LA treatment leads to S-phase cell cycle arrest in both 22Rv1 and C4-2B cell lines, and the upregulation of p21, along with the downregulation of cyclin E expression, provides insights into the molecular mechanisms underlying the growth inhibition of PCa cells by α-LA.

### α-Lipoic acid treatment induces apoptosis

**Figure 2:**
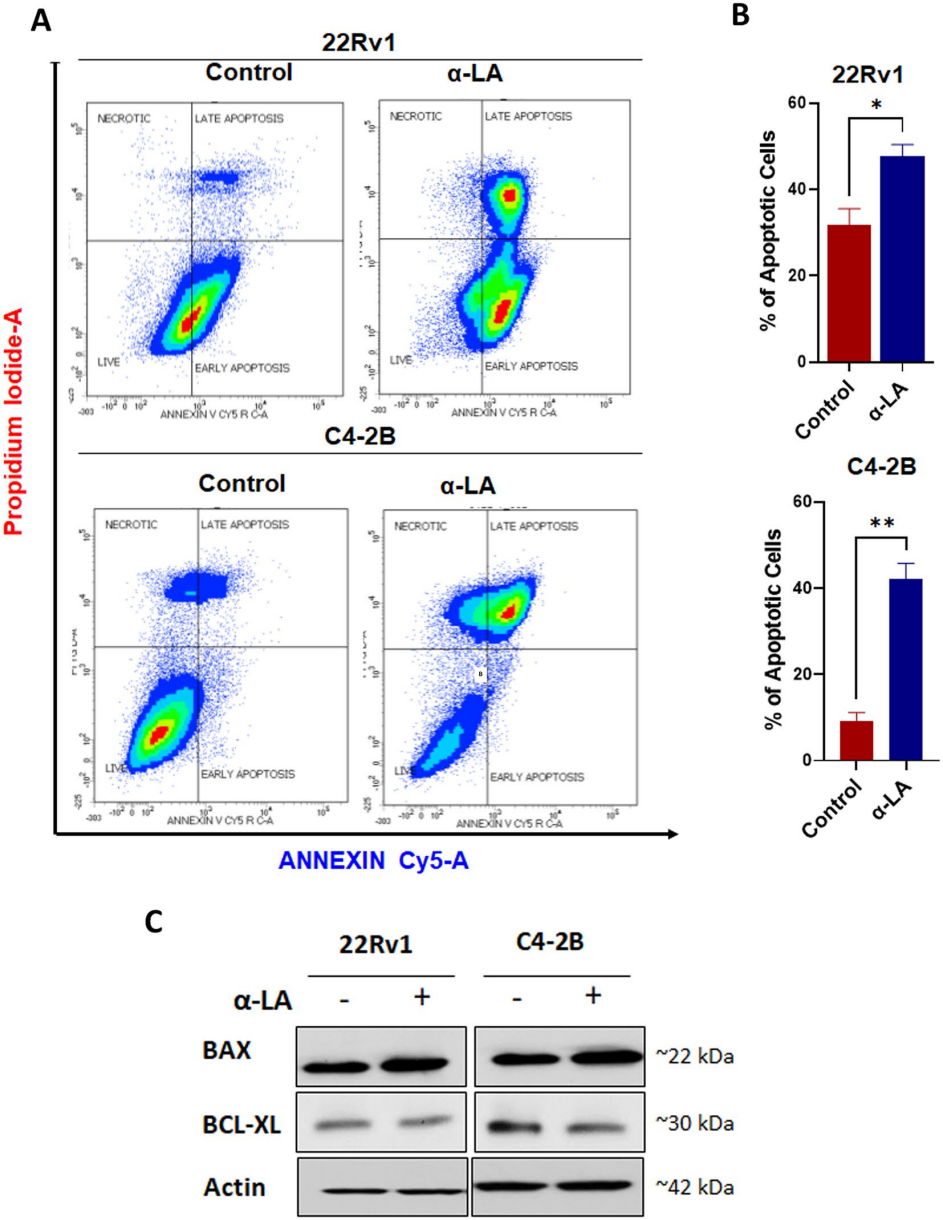
Effect of α-LA on apoptotic assay in PCa cell lines. (**A**). The percentage of viable and apoptotic cells analysis with respect to control (untreated) in 22Rv1 and C4-2B cells treated with 500 µM of α-LA for 48 h as determined by a flow cytometer of annexin-V Cy-5/PI- dual stained cells. (**B**) Quantitative analysis of these micrographs was shown as mean ± SEM. (**C**) Representative western blots of pro and anti-apoptotic genes in 22Rv1 and C4-2B cells after the treatment as described. Statistical significance was calculated using a t-test. *p < 0.05; **p < 0.001.

To ascertain whether the cell death induced by α-LA treatment followed an apoptotic pathway, we conducted flow cytometry analysis using Annexin-Cy5 staining. The flow cytometry results, as shown in Fig. 2A,B, clearly demonstrated that the α-LA-treated cells (22Rv1 and C4-2B) exhibited a significantly higher percentage (nearly 50%) of apoptosis compared to the untreated cells. Moreover, the modulation of anti-apoptotic Bcl-2 family proteins is crucial for the apoptotic process. To determine if α-LA-induced apoptosis was associated with changes in the expression profile of these proteins, we analyzed the levels of pro-apoptotic protein Bax and anti-apoptotic protein Bcl-xl^37^. α-LA treatment resulted in an increase in Bax expression and a decrease in Bcl-xl expression in both cell lines (Fig. 2C). Together, these results indicate that the modulation of Bcl-2 family proteins contributes to the apoptotic response induced by α-LA treatment.

### α-Lipoic acid induces cell death through ROS generation

**Figure 3:**
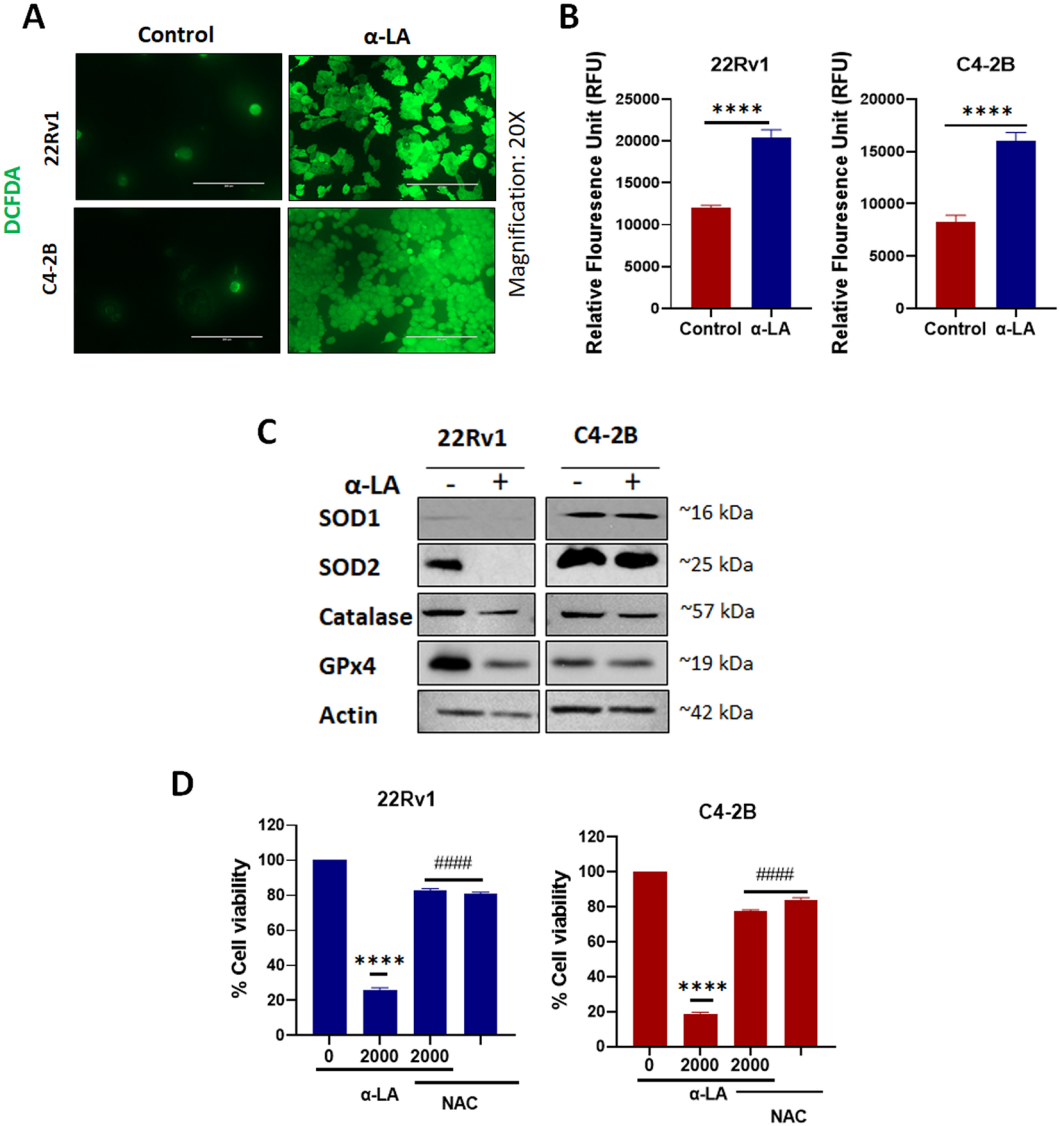
α-LA induced-ROS generation in PCa cell lines. (**A**) α-LA induced ROS generation evaluated fluorescence microscope with DCFH-DA. Scale bars = 200 µm. (**B**) ROS levels induced by α-LA were measured using a fluorescent plate reader, analyzed statistically, and presented as a bar graph as mean ± SEM. (**C**) Western blots of ROS scavenging proteins after treatment of α-LA in 22Rv1 and C4-2B as described. (**D**) Effect of pre-treatment with NAC on the α-LA-induced cell death. 22Rv1 and C4-2B cells were preincubated with 0.1 mM of NAC and then treated with 2000 µM of α-LA for 48 h. Statistical significance was calculated using a t-test. P-value ****p < 0.0001 (compared to control) or two-way ANOVA. ^####^*p* < 0.0001 (compared to 2000 µM of α-LA without NAC).

Reactive oxygen species (ROS) produced by the mitochondrial respiratory chain or from the action of NADPH oxidase (NOX) have been shown to have a crucial role in the advancement of apoptosis^38^. Images of the confocal microscopy clearly depicted that there was a significant increase in the fluorescence of DCFH-DA (green) in both 22Rv1 and C4-2B cell lines after treatment with 500 µM α-LA for 48 h (Fig. 3A,B). This indicates an elevated level of ROS in the cells treated with α-LA when compared to the control group. To better understand the mechanism underlying the enhanced ROS production following α-LA therapy, we examined the expression profiles of antioxidant enzymes-MnSOD (SOD1), Cu/ZnSOD (SOD2), catalase, and glutathione peroxidase 4 (GPx4). After 48 h of treatment with 500 µM of α-LA, we observed a reduction in the expression levels of all four enzymes in PCa cell lines (Fig. 3C). Furthermore, to investigate whether the generation of ROS induced by α-LA treatment contributes to cell death, we utilized N-acetyl cysteine (NAC), recognized as a ROS scavenger and known for increasing intracellular glutathione levels^39^. Cell viability did not change significantly when both cell lines were treated with NAC alone. However, pretreatment with 0.1 mM NAC markedly attenuated α-LA-induced cell death (Fig. 3D). These findings demonstrate that α-LA induces cell death by generating ROS, which resulted in reduced expression of anti-oxidant enzymes.

### α-Lipoic acid induces cell death via ROS-mediated HIF-1α expression and JNK/Caspase-3 signaling

**Figure 4:**
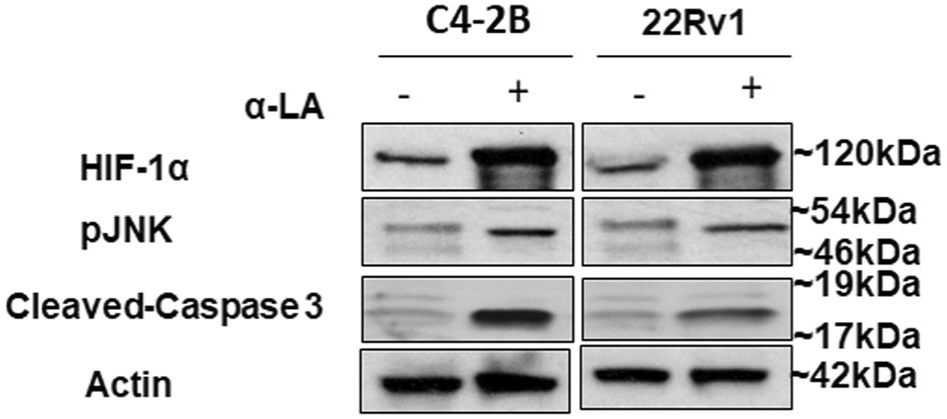
Western blot analysis showing expression of HIF-1α, pJNK, and cleaved caspase-3. The blotting depicted a higher expression of HIF-1α, pJNK, and cleaved caspase-3 upon α-LA treatment assessed in whole cell lysates isolated from control and α-LA treated 22Rv1, and C4-2B. β-actin was used as the loading control.

To investigate the impact of α-LA on apoptosis induction, we examine the expression of key proteins involved in the apoptotic pathways. Since hypoxia-inducible factor 1 alpha (HIF-1α) is essential for the physiological response to hypoxia, we measured its expression at the protein level. We observed that HIF-1α protein expression is increased in both cell lines, resulting from elevated intracellular ROS levels (Fig. 4). Further, to explore the relationship between HIF1α and caspase-3 production, we examined the expression of c-Jun N-terminal kinase (JNK) because JNK, activated by ROS, leads to apoptosis through cleavage of caspases^40^. Notably, the phosphorylated JNK (p-JNK) (Thr183/Tyr185) protein levels were markedly upregulated in cells treated with α-LA as compared to the control cells. This elevation in JNK expression correlated with the expression of cleaved-caspase-3 (Fig. 4). These results prove that α-LA induces apoptosis by activating the ROS-mediated HIF-1α expression and JNK/caspase-3 signaling pathway.

### α-Lipoic acid inhibits the PCa cell migration, invasion, and colony formation

**Figure 5:**
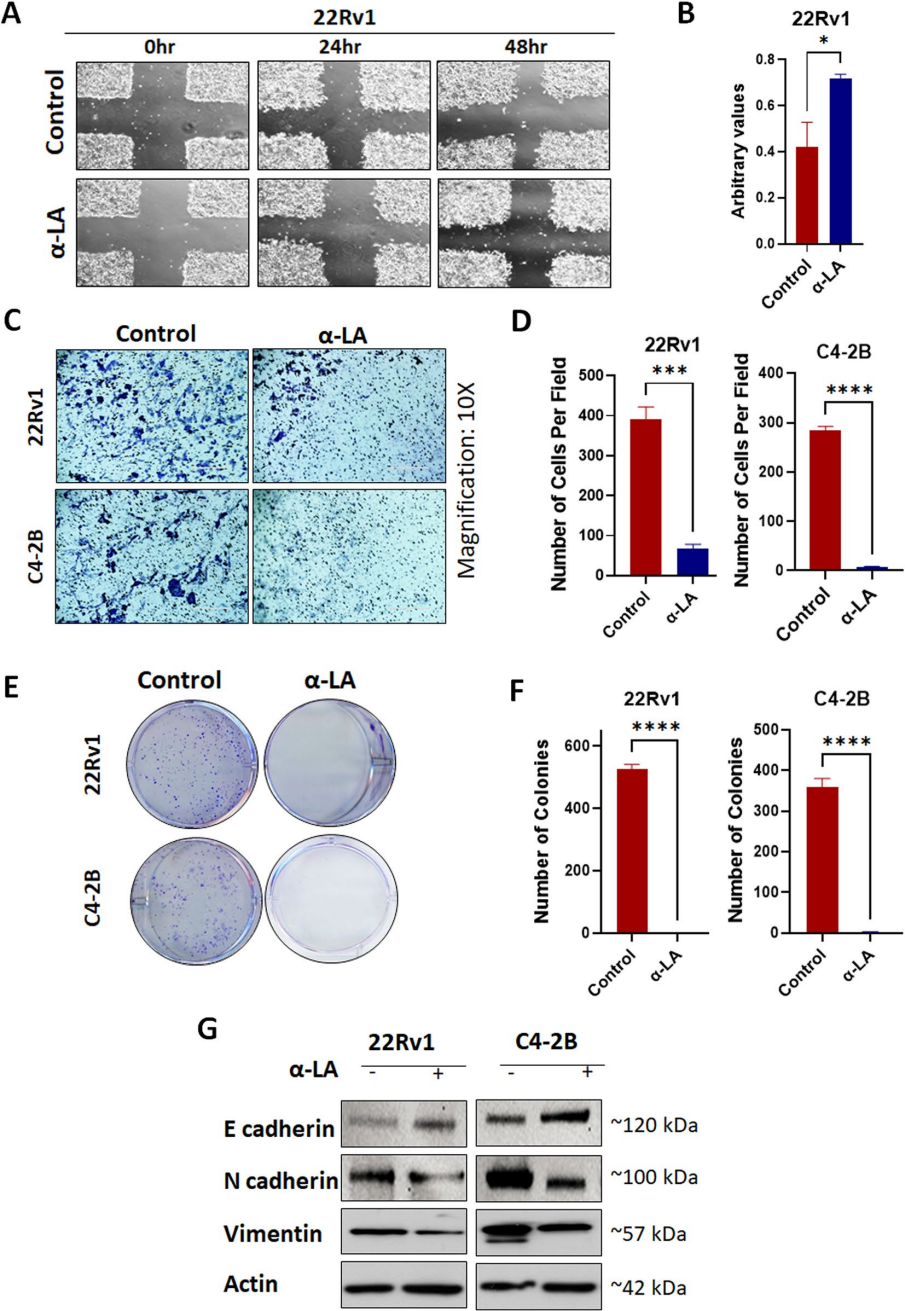
α-LA inhibited the invasion and cell migration potential of PCa cells. (**A**,**B**) Wound assay of 22Rv1 cells treated with 500 µM of α-LA for 48 h. Imaging has been done at a magnification of 10x (t = 0 h, t = 24 h & 48 h) and analyzed through Image J software. Scale bars = 400 µm. (**C**,**D**) Migration assay of 22Rv1 and C4-2B cells treated with 500 µM of α-LA for 48 h. Migrated cells were stained with crystal violet, and imaging was done in five different areas at a magnification of 10x. Stained migrated cells were counted and analyzed statistically by using a t-test. Scale bars = 400 μm. (**E**,**F**) Representative images of colony formation assay in 22Rv1 and C4-2B cells after 14–20 days of treatment with 500 µM of α-LA. Quantification was done by counting the number of colonies and represented as a bar diagram. (**G**) Western blot analysis of EMT markers after the treatment as described. Statistical significance was calculated using a t-test, and results are expressed as mean ± SEM. *p < 0.05, ***p < 0.005, ****p < 0.0001.

Migration and invasion are complex processes that are involved in the malignant transformation of tumors^41^. To investigate the effects of α-LA treatment on cell migration, scratch, and invasion assays were performed on 22Rv1 and C4-2B cell lines. We found a substantial inhibition of cell migration compared to control in 24 and 48 h, as shown in Fig. 5A,B. Similarly, transwell invasion assays revealed a consistent trend of reduced invasion in both cell lines upon α-LA treatment (Fig. 5C,D). These findings indicate that α-LA effectively inhibits PCa cells' migration and invasion capabilities.

In addition, colony formation assays were conducted to assess the impact of α-LA on the ability of cells to form colonies. After treating 22Rv1 and C4-2B cell lines with α-LA, a significant decrease in the colony-forming potential was observed (Fig. 5E,F). These results demonstrate the ability of α-LA to impair the colony formation ability of PCa cells.

To gain further insights into the molecular mechanisms underlying the migration inhibitory effect of α-LA, we examined the expression of epithelial-mesenchymal transition (EMT) markers such as E-cadherin, vimentin, and N-cadherin. Remarkably, treatment with 500 µM α-LA resulted in a significant increase in E-cadherin expression, along with decreased levels of vimentin and N-cadherin (Fig. 5G). These changes indicate a reversal of the EMT process, associated with reduced migratory and invasive capabilities of cancer cells.

### α-Lipoic acid treatment on PCa-mediated osteoblastic function

The interaction between the bone microenvironment and cancer cells results in a vicious cycle that leads to bone abnormalities and promotes tumor growth. Osteoblasts play a crucial role in bone formation, and their activity is particularly significant in PCa patients^42^. Additionally, 22Rv1 is known to form osteoblastic and osteoclastic lesions on the bone to promote bone metastasis under physiological conditions^43^. Therefore, we investigated the effect of α-LA on preosteoblastic MC3T3-E1 differentiation in the presence of a tumor microenvironment. MC3T3-E1 were cultured for 48 h using different ratios of 22Rv1-derived CM (20, 40, and 50% in αMEM) under osteoblast-differentiating conditions (in the presence of ascorbic acid and β-glycerophosphate) along with the addition of the α-LA.

**Figure 6:**
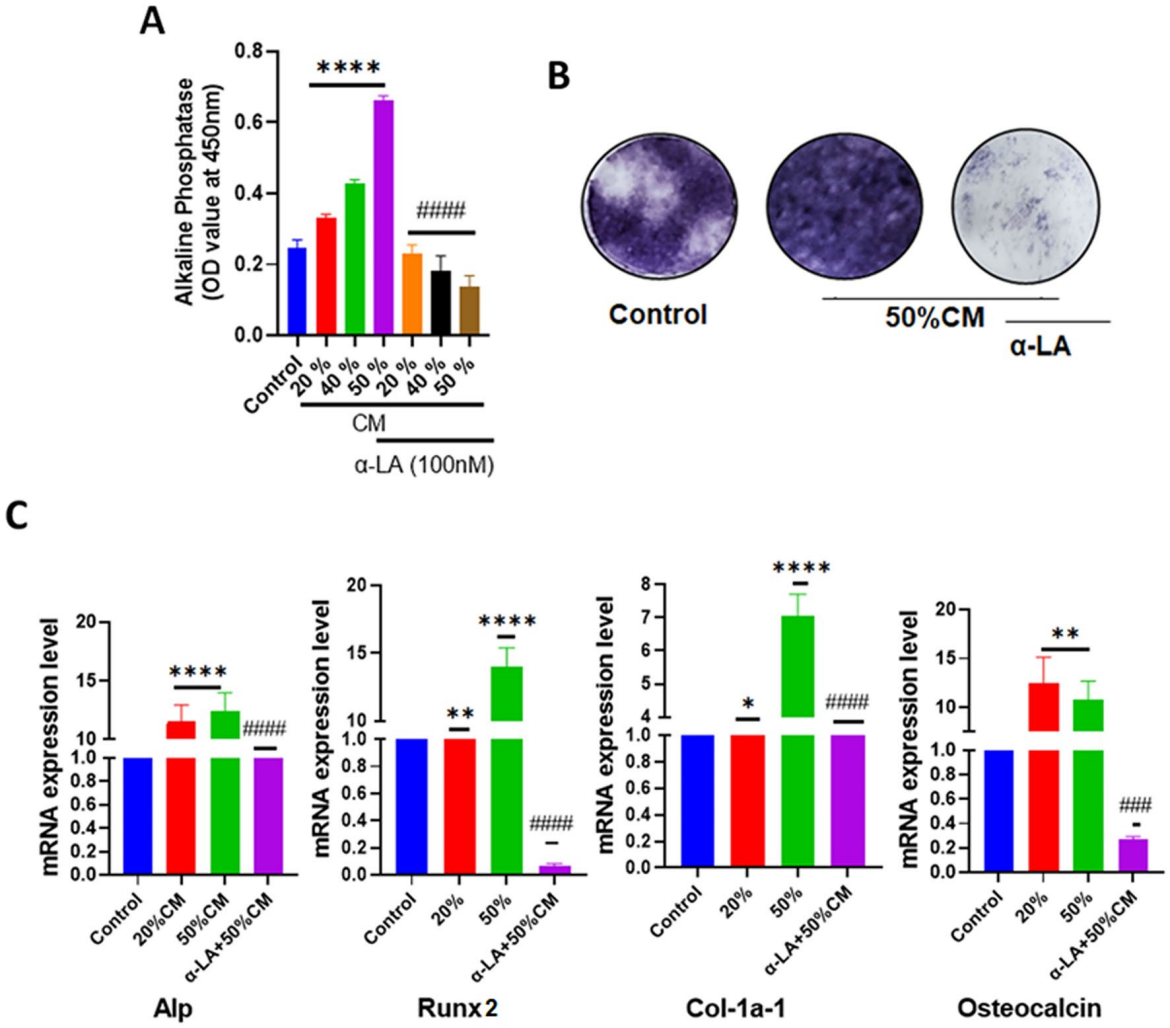
α-LA treatment inhibits PCa-mediated osteoblast differentiation. (**A**) PCa interaction with bone cells promotes osteoblast differentiation, which was inhibited by treatment with 100 nM of α-LA. Undifferentiated MC3T3-E1 cells were treated with various proportions of 22Rv1 condition medium (20%, 40%, 50%, and 50% with 100 nM of α-LA) for 48 h and subjected to alkaline phosphatase activity. (**B**) Representative ALP staining of MC3T3-E1 cells after 10 days of treatment with the alone 50% condition medium of 22Rv1 cells and 50%CM with 100 nM of α-LA. (**C**) MC3T3-E1 cells were treated with the control, 20%, 50% CM, and 50% CM with 100 nM α-LA for 48 h in the differentiation medium of osteoblast. Total RNA was harvested, RT–qPCR for osteogenic genes (alkaline phosphatase: ALP, Runx-2, Col-1a, and osteocalcin) was performed, and gene expression was normalized to that of β-actin. Statistical significance was calculated using one-way ANOVA or t-test, and results were presented as mean ± SEM, p-value; **** p < 0.0001; **p < 0.0080, *p < 0.05 (compared to control); ^####^*p* < 0.0001; ^###^*p* < 0.0003 (compared to 50%CM without α-LA).

To assess the effects of α-LA on the expression of osteoblast differentiation markers mediated by PCa, we examined the activity and staining of alkaline phosphatase (ALP). As shown by ALP activity and staining, PCa-mediated osteoblast differentiation was significantly increased compared to the control group. However, treatment with 100 nM of α-LA exhibited notable inhibition in ALP activity and staining (Fig. 6A,B). Consistent with the results of ALP activity and staining, gene expression analysis also revealed an upregulation of mRNA levels of the early osteogenic genes *Alp*, *RUNX-2,* as well as late osteogenic genes *Col-1a-1* and *OSX* in osteoblasts treated with the CM of 22Rv1 cells compared to the control. However, the presence of 100 nM of α-LA significantly decreased the expression of these genes (Fig. 6C). These findings demonstrate that α-LA effectively prevents the PCa-mediated osteoblastic function.

### α-Lipoic acid treatment on PCa-mediated osteoclast differentiation

**Figure 7:**
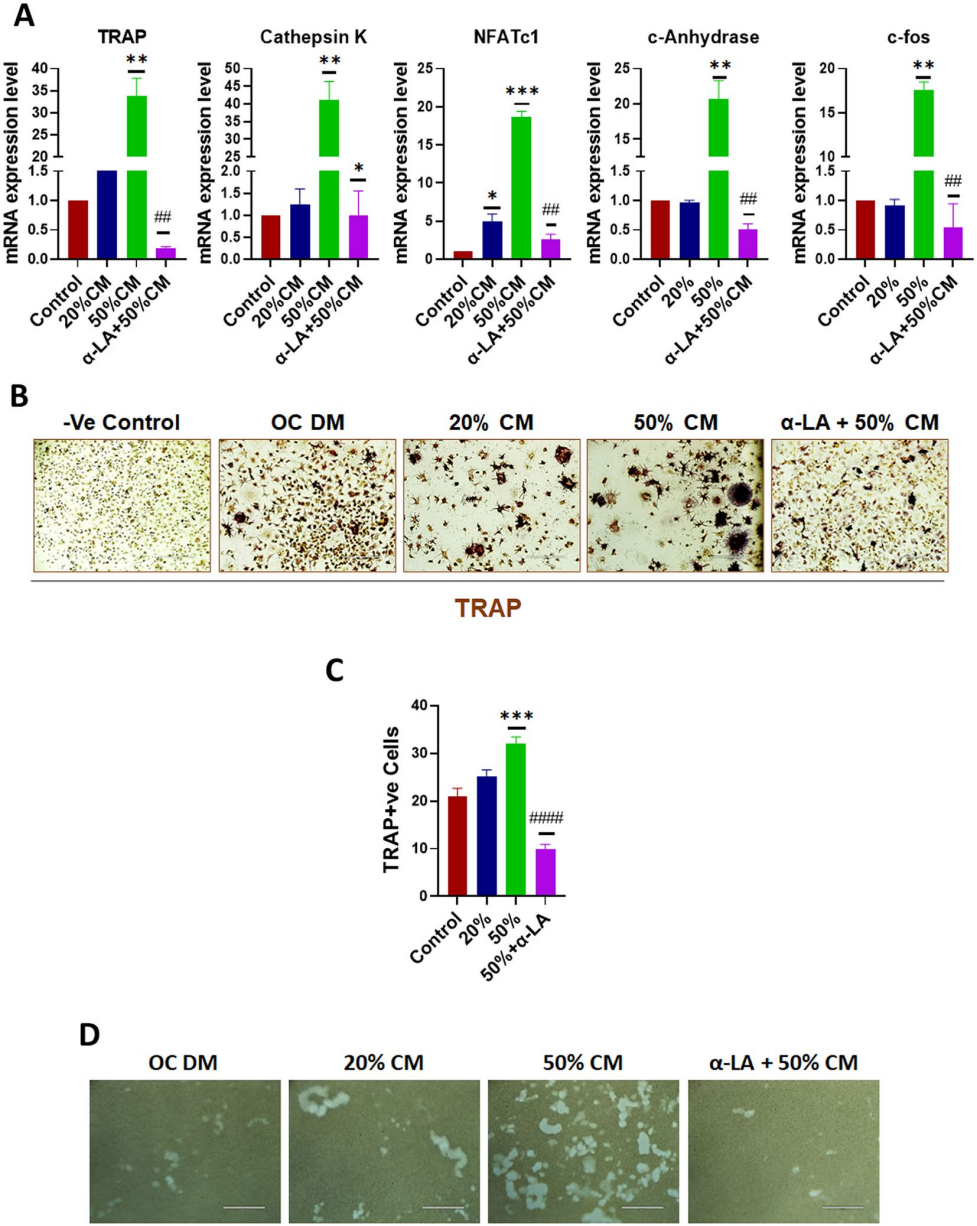
α-LA treatment inhibits PCa-mediated osteoclastic differentiation and function. (**A**) PCa interaction with bone cells promotes osteoclast differentiation, which was inhibited by the treatment with 100 nM of α-LA. BMMs from C57BL/6J mice were cultured for 7 days with M-CSF, RANKL, and various proportions of 22Rv1 condition medium (20%, 50% and 50% with 100 nM of α-LA). Total RNA was harvested, RT–qPCR for osteoclast markers (TRAP, NFATc1, carbonic anhydrase, c-fos, and cathepsin K) was performed, and gene expression was normalized to that of β-actin. (**B**) Representative TRAP staining of BMMs cells after 7 days of treatment with alone 50% condition medium of 22Rv1 cells and 50%CM with 100 nM of α-LA. (**C**) The number of TRAP + ve cells was counted and represented as a bar diagram. (**D**) Representative images of pit resorption assay of BMMs cells after 7 days of treatment as described. Statistical significance was calculated using one-way ANOVA or t-test, and results were presented as mean ± SEM, ****p < 0.0001; ***p < 0.0004; **p < 0.008; *p < 0.05 (compared to control); ^####^*p* < 0.0001; ^###^*p* < 0.0003; ^##^*p* < 0.003; ^#^*p* < 0.01 (compared to 50%CM without α-LA).

Abnormal enhancement in osteoclast differentiation is the primary factor contributing to the pathologic lesions on bone due to PCa bone metastasis^44^. Therefore, we investigated the effect of α-LA on the PCa-mediated differentiation of BMMs. qRT-PCR and TRAP (osteoclast marker) staining have been done to see the impact of α-LA on BMMs differentiation to osteoclasts (OCs). qRT-PCR data suggest that α-LA plays a crucial role in the PCa-mediated differentiation of BMMs cells. α-LA significantly decreases the PCa mediated OC differentiation genes, like *TRAP*, *CTSK* (cathepsin K), *NFATc1*, carbonic anhydrase, and c-fos (Fig. 7A). Furthermore, TRAP staining also reveals a similar observation at the differentiation of BMMs in the validation of OC differentiation and function (Fig. 7B,C).

Similarly, pit resorption assay on unique bone surfaces enabled us to observe the severity of OC activity on the bone surface environment. Single and multiple pit clusters were observed in various treatment groups (Fig. 7D). In normal OC differentiation conditions, multiple pit clusters were observed. Moreover, the BMMs were grown in 50% CM of 22Rv1, and a lot more resorbed pit clusters were formed, showing that 50% CM vigorously increased the OC formation and resorption of the osteo assay surface. Indeed, we found that α-LA (100 nM), even in the presence of 50% CM, shows an inhibitory activity for osteoclast differentiation as evident by qRT-PCR and TRAP staining. The pit resorption experiment strongly supports this observation. Collectively, α-LA in the presence of 50% CM of PCa cells significantly inhibits the OC differentiation, activity, and bone resorption.

## Discussion

In recent years, α-LA has gained attention for its potential anti-cancer properties^45^. Apart from its anti-neoplastic potential, α-LA is a necessary cofactor for various mitochondrial enzymes and plays a critical role in numerous physiological processes, notably oxidative energy metabolism^46^. Due to the antioxidant properties, α-LA has been employed to treat chronic conditions involving higher levels of oxidative stress like Alzheimer's and diabetic neuropathy. Several studies have reported the inhibitory effects of α-LA and its derivatives on tumor development and survival in various malignancies, with minimal impact on non-cancerous primary cells^47^. However, the precise mechanism of cell death induced by α-LA in transformed cell lines and cancer cells is not well established. In this study, we selected the C4-2B and 22Rv1 PCa cell lines to investigate the anti-cancer effects of α-LA. The treatment of both cell lines with α-LA had a dose-dependent decrease in cell viability, and this effect was attenuated when cells were pretreated with N-acetyl cysteine (NAC), a ROS scavenger.

Moreover, α-LA treatment led to an increase in apoptotic cell proportion and reactive oxygen species (ROS) generation. Additionally, α-LA treatment reduced the migratory, colony formation, and invasive capability of both cell lines. The expression of HIF-1α significantly increased following α-LA treatment and was comparable with the changes in ROS.

It is well known that elderly individuals, those who are more prone to developing PCa often experience severe oxidative stress related to age^48^. Excessive ROS might result in damage to important biomolecules like DNA, protein, or lipids and may act as anti-neoplastic agents, resulting in apoptotic or necrotic cellular damage^49,50^. Furthermore, tumor growth generates substantially higher oxidative stress in a hypoxic tumor microenvironment compared to normoxic circumstances. ROS in the tumor microenvironment can act as a double-edged sword because, on one end, promoting programmed cell death while also supporting cancer cell proliferation and survival by causing DNA mutations and impairing the activities of several tumor suppressor genes such as protein tyrosine phosphatases (PTPs), phosphatase and tension homolog (PTEN), and MAPK phosphatases, resulting in the activation of the PKD-NF-KB, PI3K-PKB/Akt, AND MAPK-ERK signaling cascades^51,52^. Consistent with the previous studies, which prove that oxidative stress is a medium through which α-LA exhibits apoptotic and anti-proliferative activity, it also arrests cells in the G1 phase of the cell cycle and activates p27 (kip1)-dependent cell cycle arrest in numerous malignancies and^36,46,53–55^. Our findings also demonstrate that α-LA instigated the level of ROS in both the cell lines of PCa.

Furthermore, the epithelial-mesenchymal transition (EMT) of PCa cells from the initial tumor progression results in increased metastasis and invasiveness^56^. Our findings indicate that α-LA significantly reduces the migratory potential of 22Rv1 and C4-2B cell lines, suggesting that elevated ROS levels might inhibit the migration potential of PCa cell lines. These results stand true to the previous findings, which demonstrated the cytotoxic potential of high ROS^57^. Nonetheless, HIF-1α is a crucial mediator of apoptosis expressed primarily in hypoxic conditions and allows cells to survive by stimulating survival pathways along with the upregulation of glutathione-based antioxidant genes to combat ROS production^58–60^. HIF-1α subunits have been shown to interact with the tumor suppressor p53 gene, leading to the stabilization and activation of p53 and initiation of programmed cell death in a hypoxic environment^61^. On the other hand, several studies indicate that the deletion of the p53 gene occurs owing to the stability of HIF-1α in hypoxia cells, hence reducing the initiation of apoptotic pathways^62^. Hypoxia-induced apoptosis occurs through the pro-apoptotic protein Nip3, one of the target genes of HIF-1α, which directly blocks the activity of anti-apoptotic proteins like Bcl-xl and Bcl-2^63^.

PCa patients often develop skeletal impairment due to the dissemination of primary malignant cells, which preferentially invade the bone microenvironment. Successful invasion of tumor cells requires endothelial-to-mesenchymal transitions through effector molecules like proteases and cadherins. Interestingly, our study demonstrates that α-LA treatment reduces the expression of mesenchymal markers with the increase in e-cadherin in both 22Rv1 and C4-2B cell lines at the protein level. Anatomically, the effect of PCa on bone cells can result in either osteolytic or osteoblastic phenotypes, with some cases displaying mixed lesions^64^. This is consistent with previously published studies showing that α-LA supports bone formation density by regulating the expression of genes involved in osteoblast differentiation and resorption^65,66^. Our results of ALP activity and reduced expression of key osteogenic genes signify that the α-LA has the potential to inhibit the PCa-induced osteoblastic modulation in MC3T3-E1 cells. Although the effect of prostate cancer on bone cells exhibits the osteoblastic phenotype, previously published reports established the importance of osteolytic components in the bone's progression and establishment of PCa cells^67,68^. α-LA prevents bone resorption (induced by RANKL and TNF-α) by reducing the NF-κB DNA binding in vivo^44^. Consistent with the previous reports, our data demonstrated that α-LA hampers the major osteolytic components, such as osteoclast differentiation, and their activity even in the presence of PCa-conditioned medium (22Rv1 CM) and can also protect the bone from resorption.

Overall, previous studies have proven the critical role of HIF-1α and the JNK/SAPK pathway in initiating cell death pathways^69,70^. Our findings support this observation by demonstrating higher HIF-1α and p-JNK expression along with the enhanced expression of cleaved caspase-3 following α-LA treatment in both the PCa cell lines.

In conclusion, the outcomes of the current study suggest that α-LA has significant potential in managing prostate cancer and its impact on bone cells. However, detailed mechanistic studies are needed to undercover the anti-cancer potential of α-LA responsible for suppressing the proliferation of PCa cells and, subsequently, their effect on bone cells. Furthermore, future evaluations of α-LA on prostate cancer bone metastasis animal models will provide a detailed insight into anti-metastatic potential and its effect on maintaining bone health in the presence of tumor microenvironment.

### Supplementary Information

Supplementary Information.

## Data Availability

The western blots used in this study have been submitted as a supplementary file in the original uncropped form. Other raw data supporting this study’s findings will be made available from the corresponding author upon reasonable request.
