# Supplementary figures and images for: α-lipoic acid modulates prostate cancer cell growth and bone cell differentiation
Source: Sci Rep. 2024 Feb 22;14:4404. doi: 10.1038/s41598-024-54479-x (PMC10884017; doi:10.1038/s41598-024-54479-x)

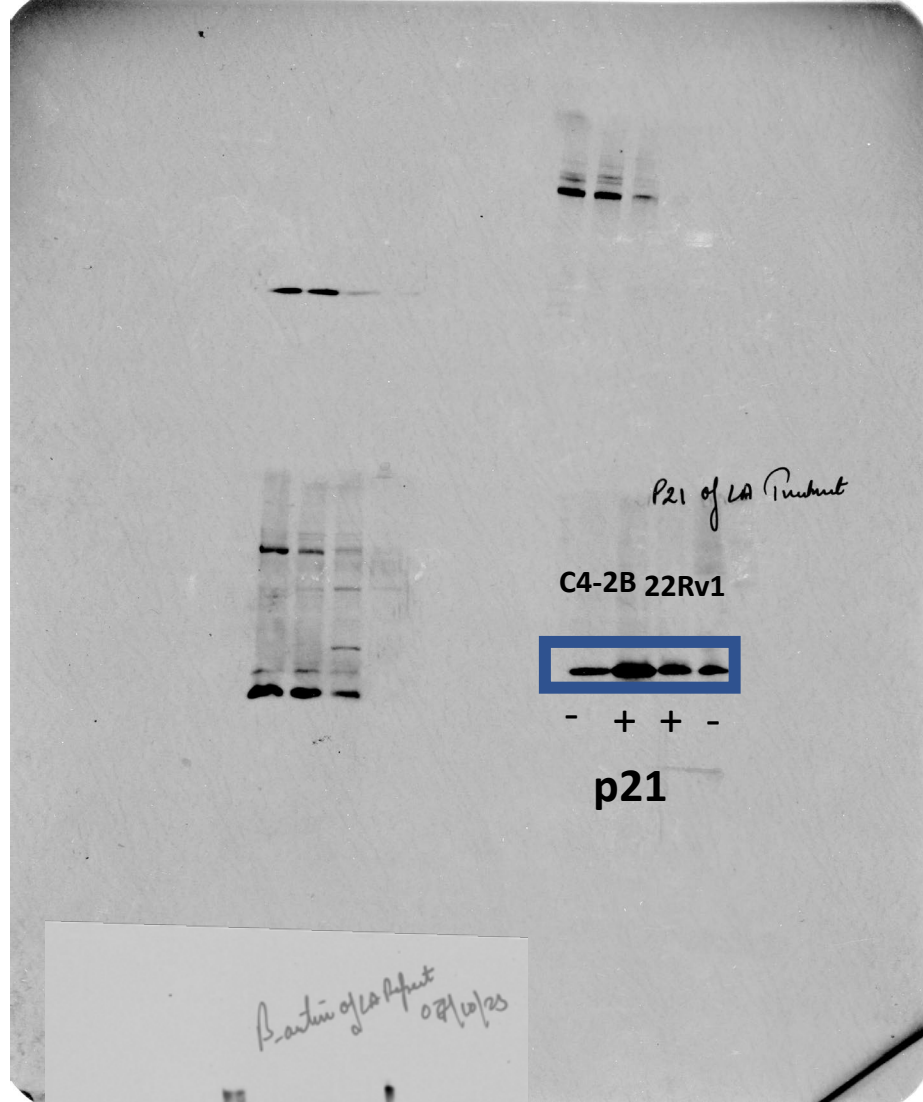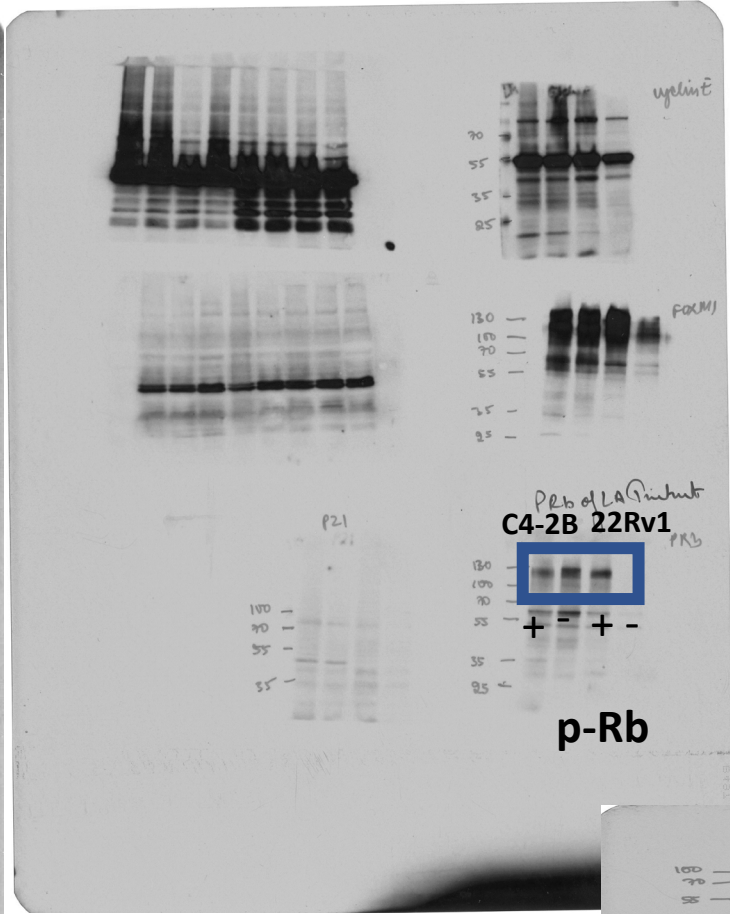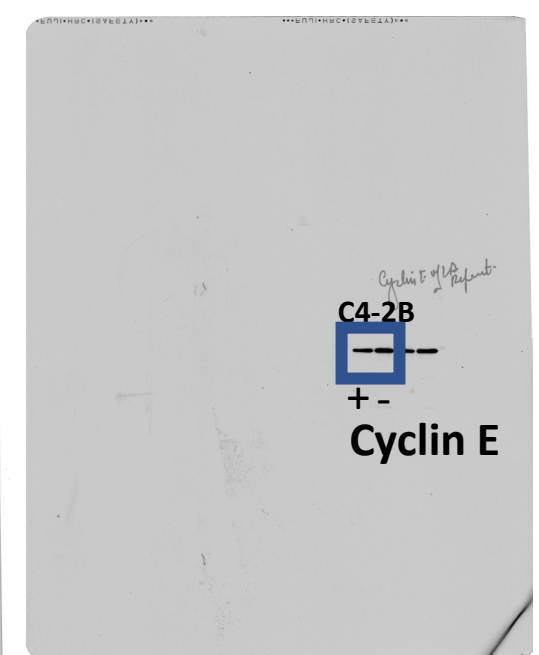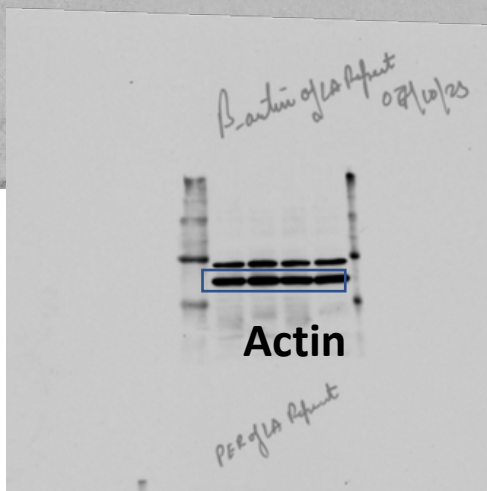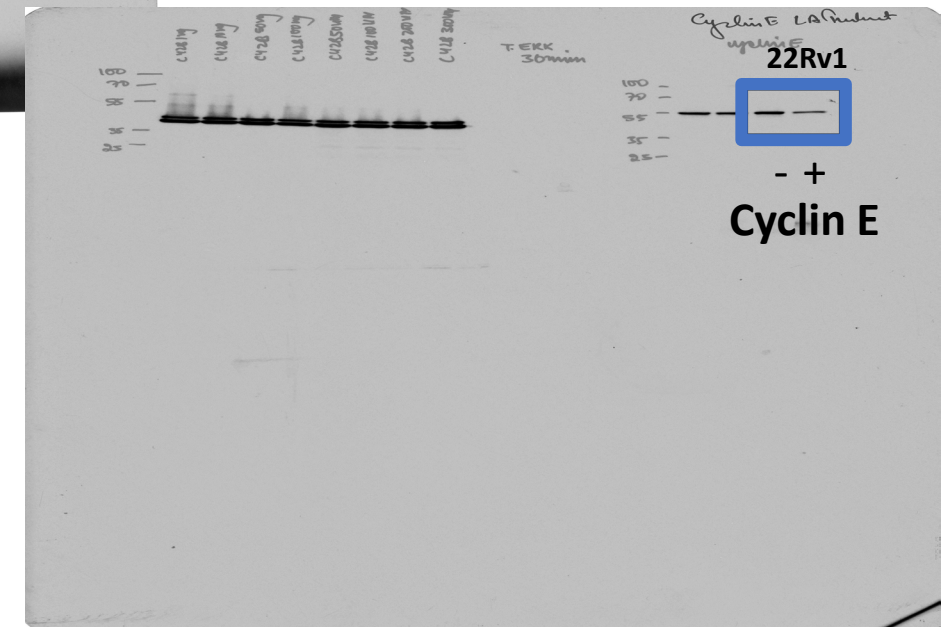

Fig1D

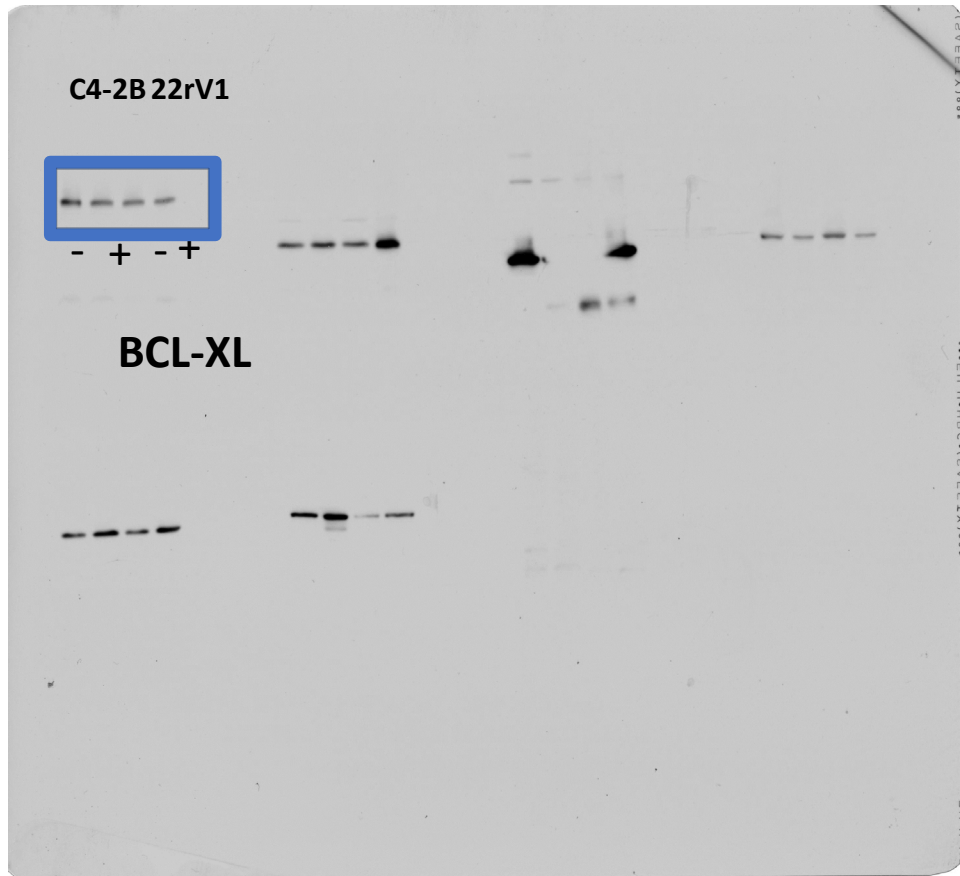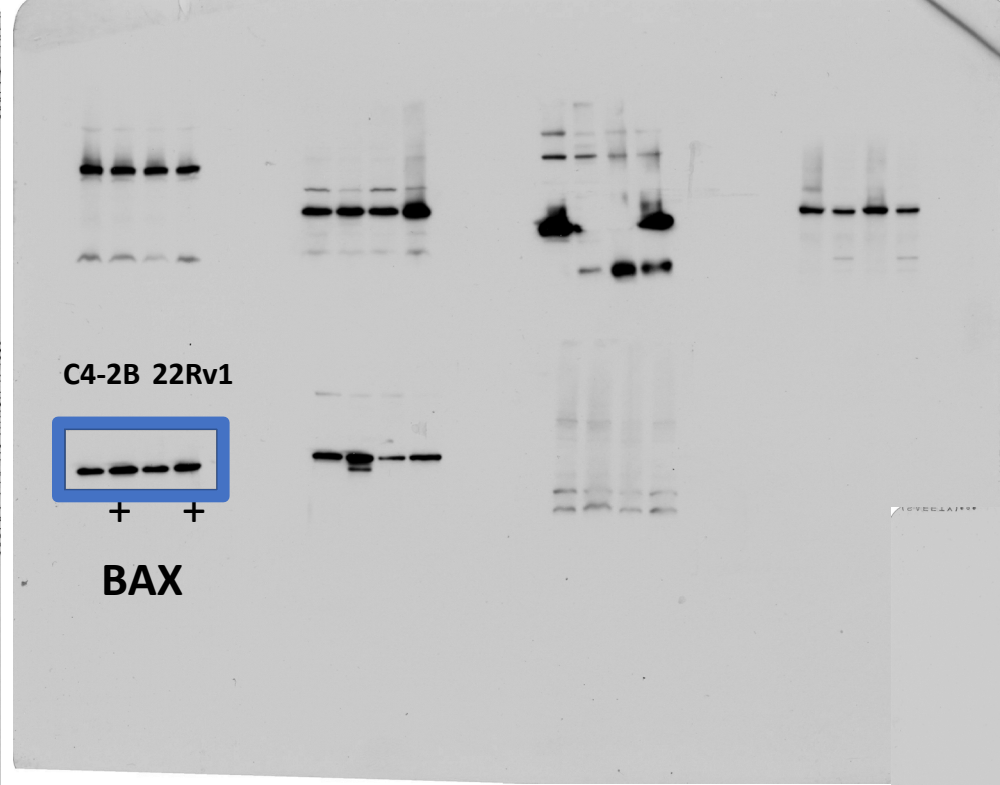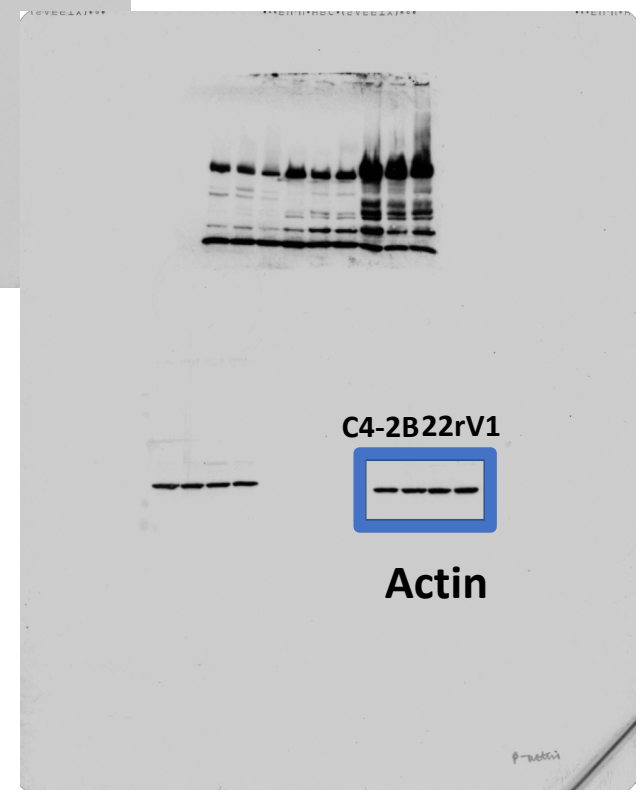

**Fig 2C**

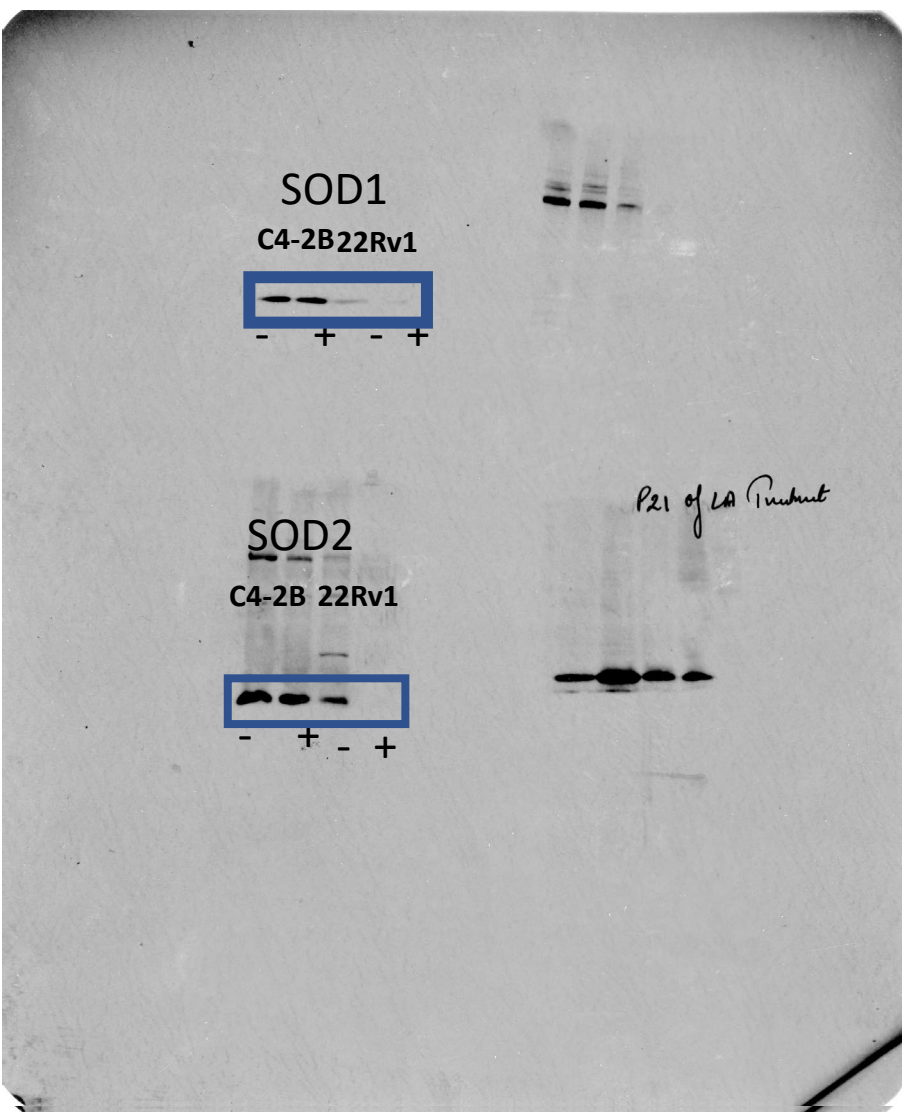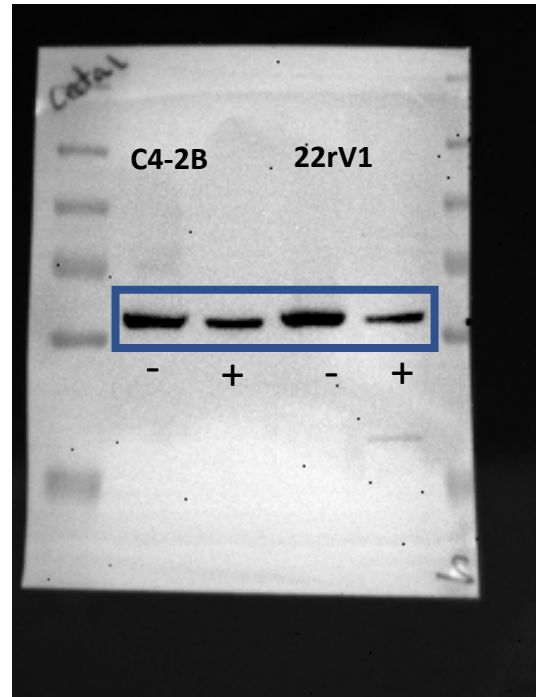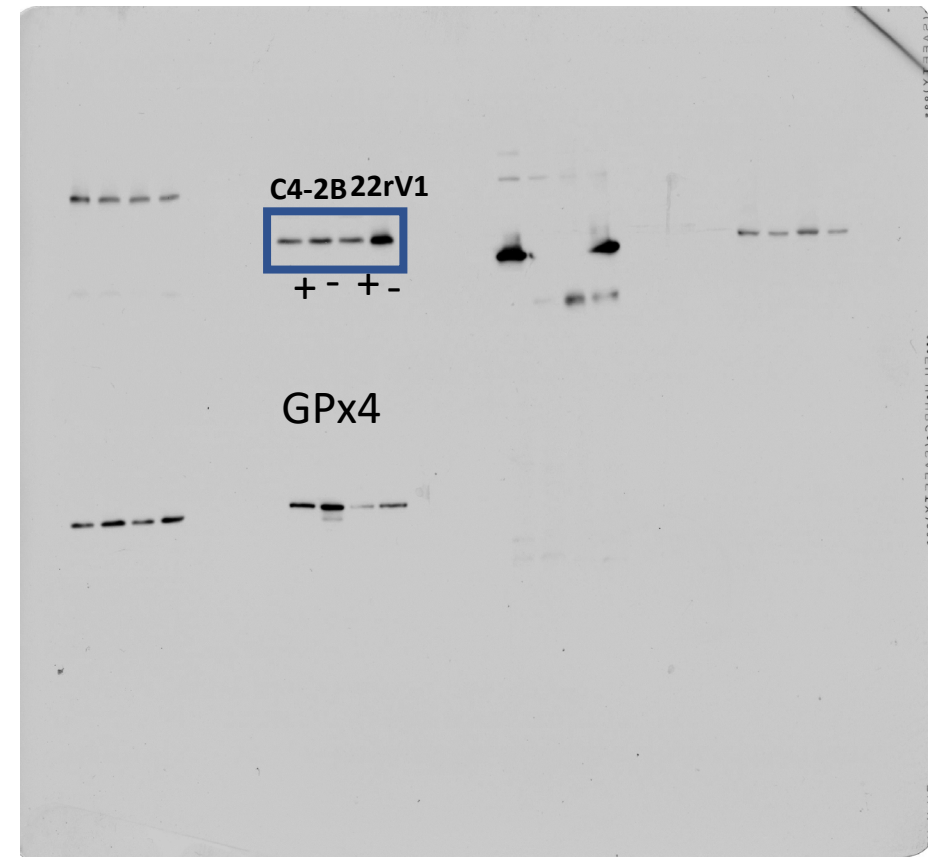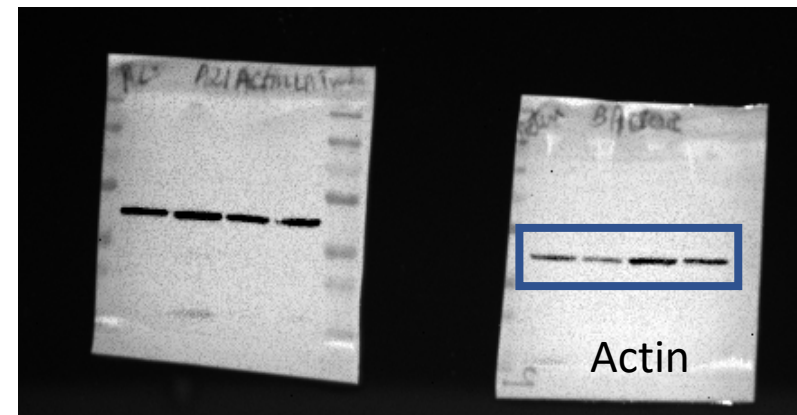

Fig3C

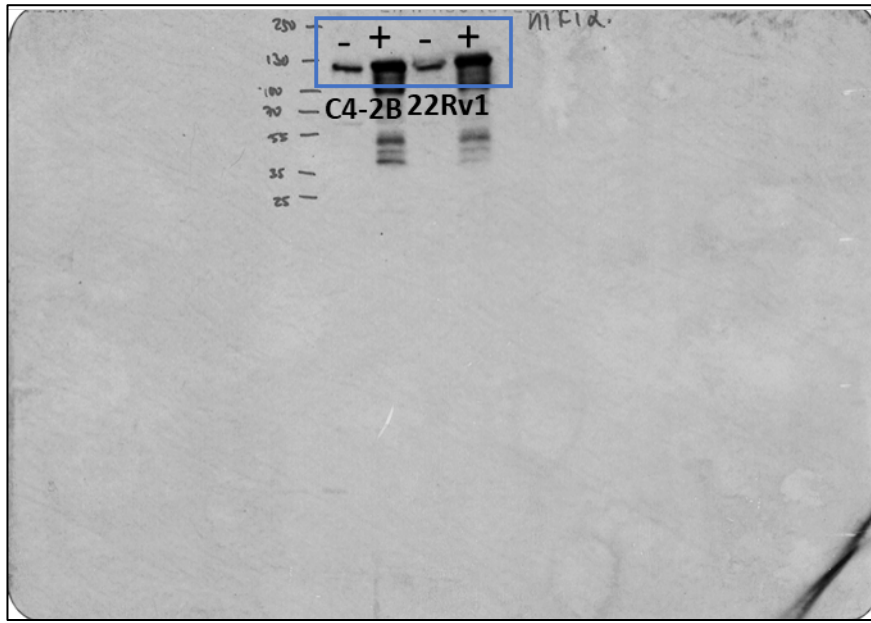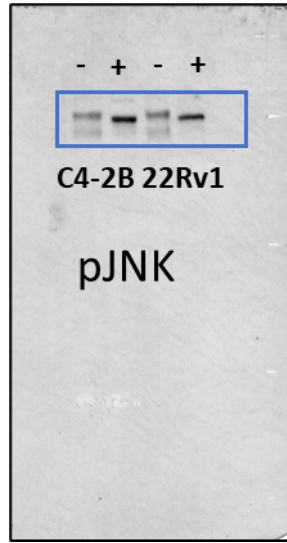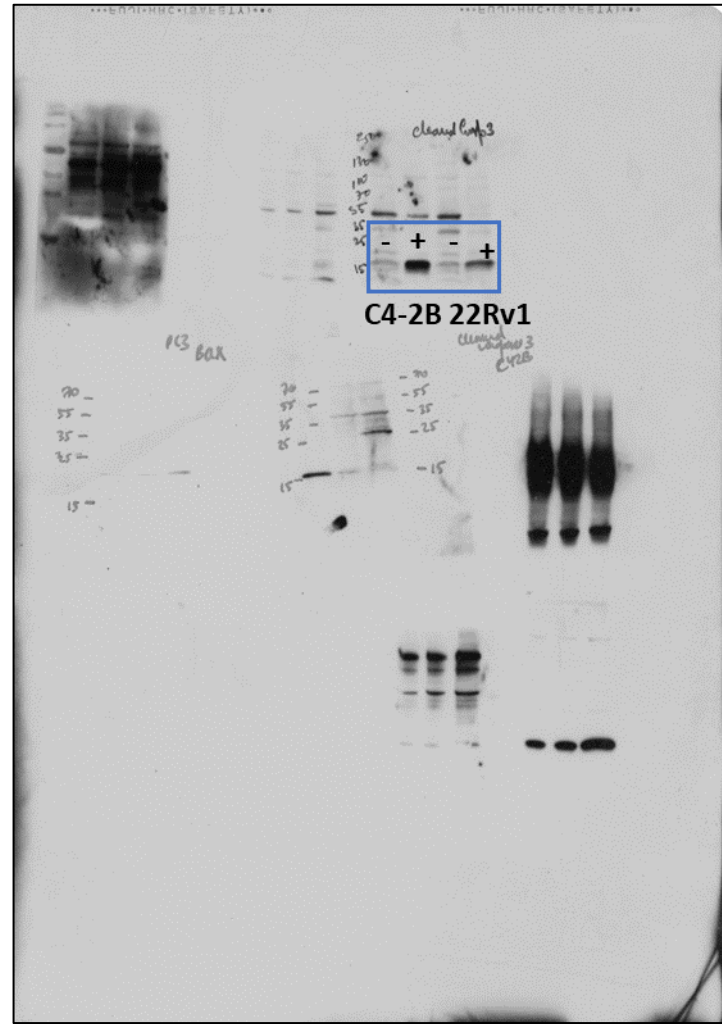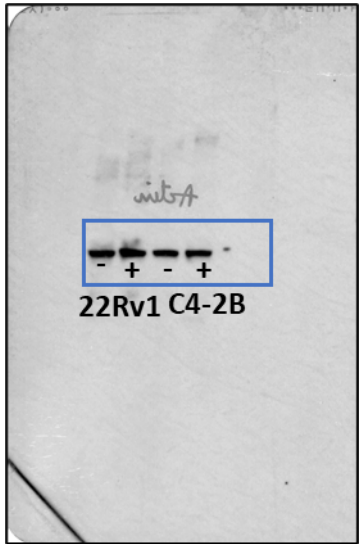

Fig4

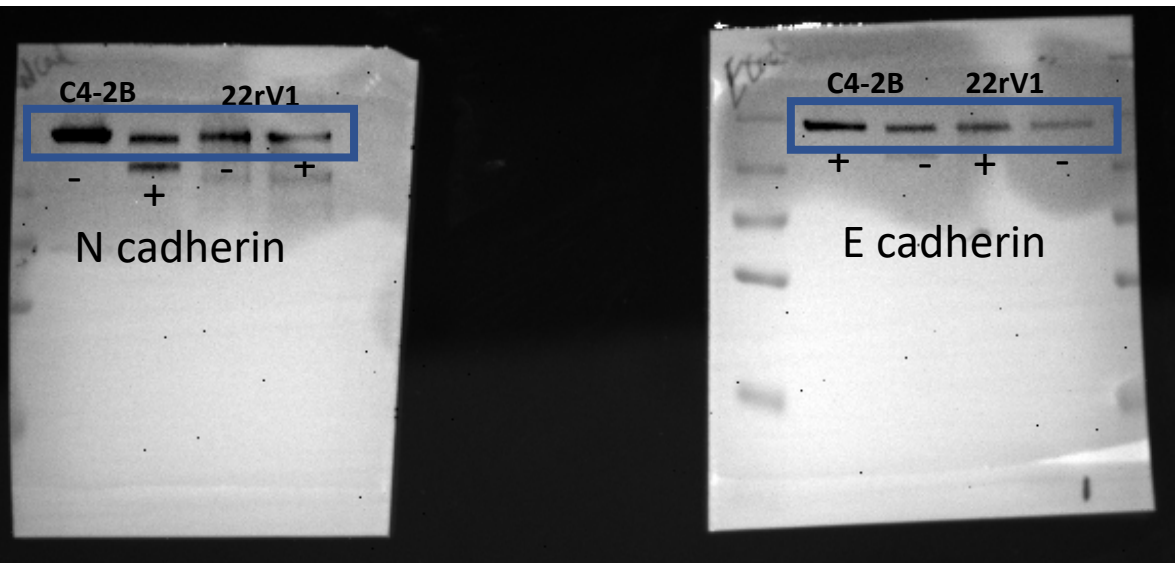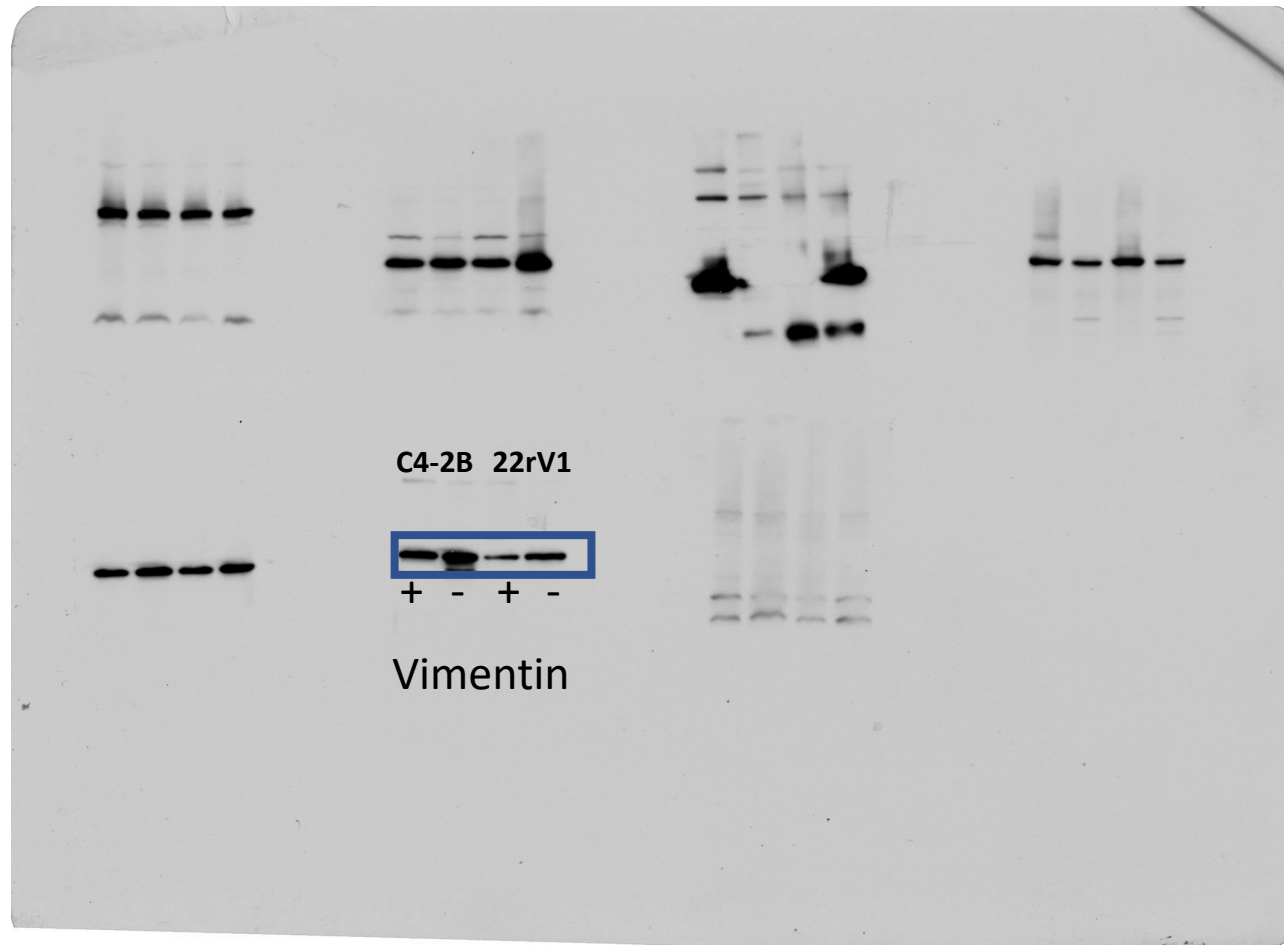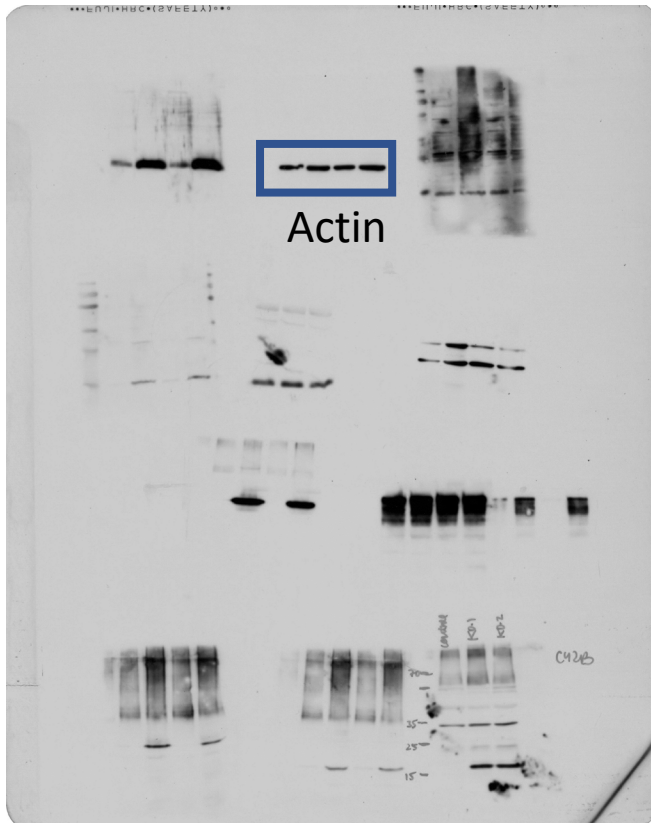

Fig5G

Supplement: Supplementary file 1 — Supplementary Information. [file 41598_2024_54479_MOESM1_ESM.pdf]
